# An Autograph Letter of Hahnemann

**Published:** 1890-11

**Authors:** 


### AN AUTOGRAPH LETTER OF SAMUEL HAHNEMANN.

Upon the opposite page we give a *fac simile* of an autograph letter by DR. SAMUEL HAHNEMANN. The original from which this copy was taken is in the possession of DR. SAMUEL LONG, of New Brunswick, New Jersey, to whose courtesy we are indebted for the opportunity to give it to the readers of THE HOMŒOPATHIC PHYSICIAN.

The letter was presented to DR. LONG by his patient, the venerable MADAME D'HERVILLY, sister-in-law of HAHNEMANN, accompanied by her own letter of which the following is the text:

"I have the pleasure and satisfaction of giving Doctor Samuel Long the accompanying letter, written to me by my brother-in-law, Doctor Samuel Hahnemann, Paris, March 12th, 1843.

"MRS. FELIX D'HERVILLY,
"NEW BRUNSWICK, July 2d, 1890."

It will be observed that the letter is in three languages: English, German, and French.

The full English translation is as follows:

(English.) "I sought truth earnestly and found it."

(German.) "Cull roses while the roses bloom,
To-morrow is not to-day;
Lose not an hour, for, ah, too soon
Time speedeth swift away."

(French.) "The most valuable treasures are an irreproachable conscience and good health; the love for God and the study of one's self gives the one, Homœopathy gives the other."

This reproduction was, in the first instance, photographed from the original, and then, by a new process, transferred to a metal plate, from which the print was made.

We are certain our readers will appreciate the opportunity to possess a *fac simile* of the handwriting of the immortal HAHNEMANN.

